# Dorsal striatum c-Fos activity in perseverative ephrin-A2A5^−/−^ mice and the cellular effect of low-intensity rTMS

**DOI:** 10.3389/fncir.2023.1179096

**Published:** 2023-06-15

**Authors:** Maitri Tomar, Jennifer Rodger, Jessica Moretti

**Affiliations:** ^1^School of Biological Sciences, University of Western Australia, Crawley, WA, Australia; ^2^Brain Plasticity Lab, Perron Institute for Neurological and Translational Science, Nedlands, WA, Australia

**Keywords:** habit formation, dorsal striatum, nucleus accumbens, rTMS, ephrin, c-Fos

## Abstract

**Introduction:**

Overreliance on habit is linked with disorders, such as drug addiction and obsessive-compulsive disorder, and there is increasing interest in the use of repetitive transcranial magnetic stimulation (rTMS) to alter neuronal activity in the relevant pathways and for therapeutic outcomes. In this study, we researched the brains of ephrin-A2A5^−/−^ mice, which previously showed perseverative behavior in progressive-ratio tasks, associated with low cellular activity in the nucleus accumbens. We investigated whether rTMS treatment had altered the activity of the dorsal striatum in a way that suggested altered hierarchical recruitment of brain regions from the ventral striatum to the dorsal striatum, which is linked to abnormal habit formation.

**Methods:**

Brain sections from a limited number of mice that underwent training and performance on a progressive ratio task with and without low-intensity rTMS (LI-rTMS) were taken from a previous study. We took advantage of the previous characterization of perseverative behavior to investigate the contribution of different neuronal subtypes and striatal regions within this limited sample. Striatal regions were stained for c-Fos as a correlate of neuronal activation for DARPP32 to identify medium spiny neurons (MSNs) and for GAD67 to identify GABA-ergic interneurons.

**Results and discussion:**

Contrary to our hypothesis, we found that neuronal activity in ephrin-A2A5^−/−^ mice still reflected the typical organization of goal-directed behavior. There was a significant difference in the proportion of neuronal activity across the striatum between experimental groups and control but no significant effects identifying a specific regional change. However, there was a significant group by treatment interaction which suggests that MSN activity is altered in the dorsomedial striatum and a trend suggesting that rTMS increases ephrin-A2A5^−/−^ MSN activity in the DMS. Although preliminary and inconclusive, the analysis of this archival data suggests that investigating circuit-based changes in striatal regions may provide insight into chronic rTMS mechanisms that could be relevant to treating disorders associated with perseverative behavior.

## 1. Introduction

Repetitive transcranial magnetic stimulation (rTMS) is a non-invasive brain stimulation technique that is currently approved for the treatment of depression (O'Reardon et al., 2007) and obsessive compulsive disorder (OCD) (Carmi et al., 2018). However, the mechanisms of how rTMS achieve therapeutic outcomes are not well understood, and increased understanding can be useful in refining its use. Currently, we know that rTMS can modulate neuronal activity (Aydin-Abidin et al., 2008; Moretti et al., 2022) and the activity of various neurotransmitters, including dopamine (Keck et al., 2002; Moretti et al., 2020), which is thought to contribute to rTMS-induced changes.

Previously, a transgenic ephrin-A2A5^−/−^ mouse strain has been used in conjunction with rTMS to measure structural and functional plasticity induced within abnormal neural pathways. Ephrins are membrane-bound ligands of Eph tyrosine kinase receptors and are important in cell migration and axon guidance during development (Wilkinson, 2001). As a result, ephrin- A2A5^−/−^ mice, which lack the ephrin-A2 and ephrin-A5 ligands, show disorganized axonal projections in neuronal circuits throughout the brain due to disrupted Eph/ephrin signaling. These mice display abnormal visual topography and visuomotor behavior which are both partially rescued by low-intensity rTMS (LI-rTMS) (Rodger et al., 2012; Makowiecki et al., 2014; Poh et al., 2018). In addition, the mice show reduced dopaminergic innervation of the striatum (Sieber et al., 2004; Cooper et al., 2009) associated with abnormal behavioral patterns in goal-directed behavior (Poh et al., 2018), but the effects of LI-rTMS on this phenotype are less well understood.

A previous study (Moretti et al., 2021) explored whether the different goal-directed behaviors of ephrin-A2A5^−/−^ mice, compared to wildtype mice, were due to abnormal motivation processing, by comparing the performance of both strains in progressive ratio (PR) tasks. PR tasks are a good measure of motivation as they involve an increasing instrumental response requirement for a reward. Highly motivated animals will continue to respond consistently, while animals with low motivation stop or slow their response (Hodos, 1961; Aberman et al., 1998). The potential ameliorating effect of chronic excitatory LI-rTMS delivered during the PR task was also examined. Unexpectedly, the results did not support a motivation phenotype: ephrin-A2A5^−/−^ mice showed perseverative behavior in the PR task, with a greater number of responses compared to wildtype mice despite increasing task difficulty (Moretti et al., 2021). Compared to wildtype mice, ephrin-A2A5^−/−^ mice also showed reduced c-Fos expression in the nucleus accumbens (NAc) after 2 weeks' performance on the PR task (Moretti et al., 2021).

It had been suggested that the behavioral phenotype of perseverative responding and reduced accumbal activity in ephrin-A2A5^−/−^ mice may reflect an accelerated dorsal shift in neuronal activity characteristic of habitual behavior (Segovia et al., 2012). The behavioral shift from goal-directed to habitual behavior involves dopaminergic pathways and hierarchical recruitment of brain regions from the ventral striatum to the dorsal striatum, which would correspond to the reduced activity in the nucleus accumbens (ventral striatum) in ephrin-A2A5^−/−^ mice (Segovia et al., 2012; Liu et al., 2013).

Interestingly, chronic excitatory LI-rTMS delivered to ephrin-A2A5^−/−^ mice during the PR task did not significantly alter behavior but did ameliorate the reduced c-Fos expression in the ventral striatum of ephrin-A2A5^−/−^ mice, resulting in expression that was similar to wildtype levels (Moretti et al., 2021). Therefore, in ephrin-A2A5^−/−^ mice, LI-rTMS may mitigate abnormal activity along the mesolimbic pathway associated with behavioral inflexibility and habit. One possibility is that it delays or interferes with an accelerated shift in neuronal activity toward the dorsal striatum.

However, Moretti et al. (2021) did not include the analysis of the c-Fos activity in the dorsal striatum. Therefore, in this study, we aim to understand whether (1) the altered dorsal striatum activity occurred in ephrin-A2A5^−/−^ mice, which could partially explain the perseverative behavior in these mice and (2) whether LI-rTMS treatment altered the dorsal striatal activity in ephrin-A2A5^−/−^ mice. In this study, we used the archival tissue from the study by Moretti et al. (2021) to identify and compare the activation of neuronal populations across the entire striatum, investigating the dorsomedial striatum (DMS), dorsolateral striatum (DLS), and nucleus acccumbens (NAc) in wildtype and ephrin-A2A5^−/−^ mice that either received active rTMS or sham stimulation, using c-Fos immunohistochemistry. Due to the archival nature of this tissue, our sample size is not sufficient to warrant firm conclusions, and without multiple timepoints, we cannot definitively demonstrate a change in the progression of hierarchical recruitment. However, we present these data as supporting evidence to Moretti et al. (2021) to determine, at least at this single timepoint, whether activity in the striatum reflects a relative difference in striatal activity contribution. This brief report acts as preliminary data to support whether the hypothesis that rTMS alters circuit function and/or hierarchical recruitment of activity has any potential for future research, especially research interested in the transition from goal-directed to habitual behavior, such as drug addiction (Everitt and Robbins, 2005) and OCD (Gillan et al., 2011). However, future research requires greater sample sizes and multiple time points to fully characterize such changes.

## 2. Materials and methods

### 2.1. Animal tissue

**Figure 1:**
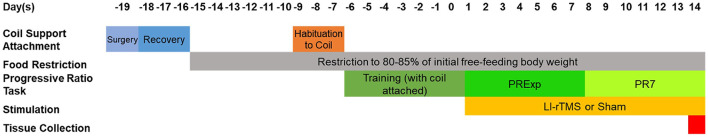
Outline of the experimental paradigm performed by Moretti et al. (2021). Reproduced from Moretti et al. (2021) Copyright 2021, with permission from Elsevier. http://www.sciencedirect.com/journal/behavioural-brain-research.

All experiments were approved by the University of Western Australia Animal Ethics Committee (AEC 100/1639). Sagittal mouse brain sections (40 μm) from 11 adult (8–24-week-old) wildtype C57BL6/J mice (sham: 6 mice (2 male and 4 female mice); rTMS: 5 mice (2 male and 3 female mice) and 10 ephrin-A2A5^−/−^ mice [sham: 6 (4 male and 2 female mice); rTMS: 4 mice (1 male and 3 female mice)] (Moretti et al., 2021) were used. The ephrin-A2A5^−/−^ mice were backcrossed onto C57BL6/J mice for >20 generations, bred from heterozygous ephrin-A2^−/−^A5^+/−^ parents and genotyped at weaning (Feldheim et al., 2000). In the previous study, following habituation to the LI-rTMS coil and training in the operant box, mice performed 1 week of an exponential PR task (required responses increase under an exponential equation) followed by 1 week of PR7 (requirement started at 7 responses and increased by 7 with each reinforcement) (Moretti et al., 2021). Mice received 14 days of biomimetic high-frequency stimulation (BHFS) LI-rTMS or sham during the first 10 min of the daily PR task and were euthanized 90 min after the beginning of the final PR task to correspond with the peak of c-Fos expression (Moretti et al., 2021). A summary of the experimental design from Moretti et al. (2021) is reproduced in Figure 1. The previous study showed perseverative behavior in both treated and untreated ephrin-A2A5-/- mice, as well as low c-Fos expression in the NAc of untreated mice, which showed improvement following LI-rTMS (Moretti et al., 2021). It was speculated that the low c-Fos expression in the NAc may reflect an accelerated shift from goal-directed to habit in untreated ephrin-A2A5^−/−^ mice, which could have been delayed at a cellular level by LI-rTMS (Moretti et al., 2021). However, in the previous study, neuronal activation in the dorsal striatum was not investigated. In the current study, we used additional tissues from the same animals as Moretti et al. (2021) to confirm the shift in neuronal activity from regions involved in goal-directed behavior to form a habit and whether LI-rTMS delayed it at a cellular level.

### 2.2. Immunofluorescence

Every fifth sagittal brain section which contained the dorsal striatum was identified using the mouse brain atlas (Paxinos and Franklin, 2012). These sections were stained for three different antibodies: c-Fos (rabbit polyclonal c-Fos antibody, 1:5000, Abcam, ab190289), a marker for neuronal activity (Bullitt, 1990); cAMP-regulated phosphoprotein-32 kD (DARPP32) (purified mouse anti-DARPP32, 1:2000, BD Transduction Laboratories, 611520), a marker for MSNs (Anderson and Reiner, 1991); and glutamate decarboxylase 67 (GAD67) (anti-Goat, 1:750, R&D systems, AF2086-SP), a marker for GABAergic neurons (Lazarus et al., 2015).

Free-floating sections were washed 3 × in PBS (5 min each), permeabilized by washing in 0.1% Triton-X in PBS (PBS-T) for 15 min, and incubated for 2 h in a blocking buffer of 2% bovine serum albumin (Sigma) and 3% normal donkey serum (Sigma) diluted in 0.1% PBS-T. Then, sections were incubated with primary antibodies in a blocking buffer overnight at 4°C. Sections were washed in PBS-T 3 × (10 min each) and incubated for 2 h with secondary antibodies diluted in blocking buffer to 1:600 (donkey anti-rabbit IgG Alexa Fluor 488, Invitrogen, Thermo Fisher, A21206; donkey anti-mouse IgG Alexa Fluor 555, Invitrogen, Thermo Fisher, A21202; and donkey anti-goat IgG H&L Alexa Fluor 647, ab150131). Sections were then washed in PBS-T 3 × (10 min each) and incubated for 10 min in Hoescht in PBS (1:1000, Invitrogen). Finally, the sections were washed in PBS 3 × (10 min each) and mounted on gelatin-subbed slides, coverslipped with a mounting medium (Dako, Glostrup, Denmark), and sealed with nail polish. Slides were stored at 4°C in a light-controlled environment until imaging.

### 2.3. Imaging and quantification

For accurate locations of DMS and DLS in sagittal sections, we used the Chon et al. (2019) atlas annotated onto a 3D magnetic resonance imaging (MRI) data of the P56 mouse brain atlas using the software ITK-SNAP (Yushkevich et al., 2006). Sections were imaged through z-stacks (2 μm apart) on a Nikon C2 confocal microscope (Nikon, Tokyo, Japan) at 40 × magnification. During imaging, sections from one ephrin-A2A5^−/−^-rTMS animal were excluded due to poor staining (ephrin-A2A5^−/−^-rTMS *n* = 3). Cell counts were performed using stereological principles using ImageJ software and were carried out blinded to the experimental group after all images were captured.

**Figure 2:**
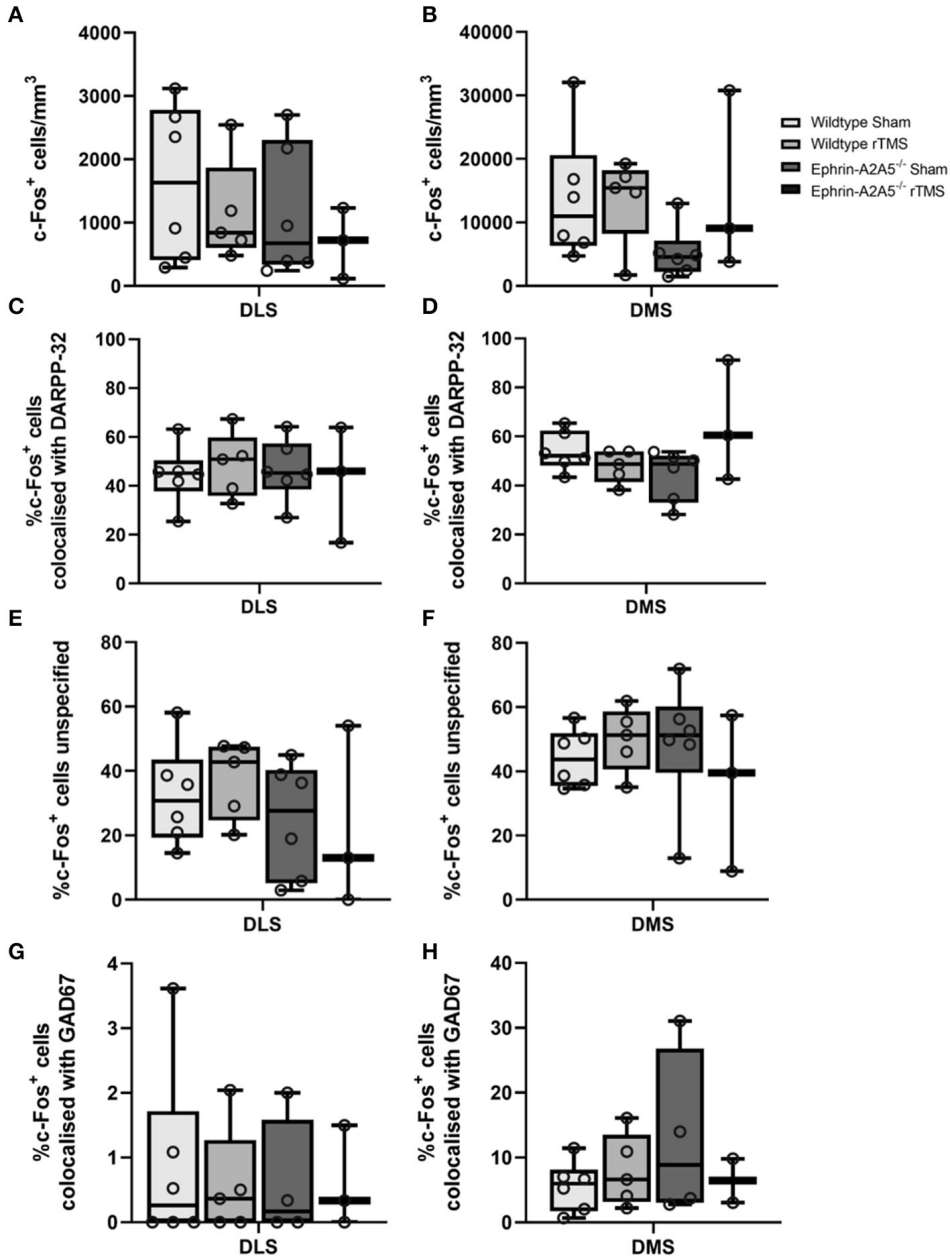
c-Fos activation in DMS and DLS across rTMS treated and untreated wildtype and ephrin-A2A5^−/−^mice. Total c-Fos density (cells/mm^3^) **(A, B)**; % of c-Fos^+^ cells colocalized with DARPP32^+^ cells **(C, D)**; unspecified c-Fos^+^ cells **(E, F)**; and c-Fos^+^ cells colocalized with GAD67^+^-DARPP32^−^ **(G, H)**. Points represent individual animal counts. Overall, c-Fos activation did not differ between strains or treatment groups. However, the significant omnibus test indicated a difference in DMS c-Fos-DARPP32 colocalization. Results suggested that in ephrin-A2A5^−/−^, rTMS increased the percentage of c-Fos-DARPP32 colocalization but pairwise *post hoc* comparisons did not survive multiple corrections.

The total number of c-Fos single-labeled cells, c-Fos^+^/DARPP32^+^ double-labeled cells, and c-Fos^+^/GAD67^+^/DARPP32^−^ double-labeled cells were identified and counted for each image (Figure 2). The combination of DARPP32^+^ and GAD67^+^ double labeling was not assessed since DARPP32^+^ cells are also GAD67^+^ because they are GABAergic in nature. c-Fos^+^ cells not colocalized with DARPP32 were classified as unspecified c-Fos.

### 2.4. Statistical analysis

For analysis, the total c-Fos cells/mm^3^, the percentage of c-Fos cells colocalized with DARPP32, and GAD67 were calculated for each animal.

Assumptions of normality and homogeneity were checked. For DLS counts, the assumptions of normality were not met for percentage c-Fos-GAD67 and total c-Fos cells/mm^3^ counts (*p* < 0.05). The homogeneity of variance was checked, and it was met for all datasets (Levene's test, *p* > 0.05). For DMS counts, the assumptions of normality were not met for percentage c-Fos-DARPP32, percentage c-Fos-GAD67, and total c-Fos cells/mm^3^ (*p* < 0.05). The assumption of homogeneity of variance was not met for percentage c-Fos-DARPP32 data (Levene's test, *p* < 0.05) but was corrected with logarithmic transformation.

To compare whether cell counts changed across groups, multiple two-way analyses of variance (ANOVAs) were used with the independent variables of strain and treatment. Each ANOVA had the dependent variable of either total c-Fos cells/mm^3^, percentage c-Fos-DARPP32, or percentage unspecified c-Fos cell count data and was run separately for both DLS and DMS. ANOVA was also performed for c-Fos^+^/GAD67^+^/DARPP32^−^ cell counts for the DLS region. For the DMS regions, due to a small sample size, the ephrin-rTMS group was excluded from statistical analysis for c-Fos^+^/GAD67^+^/DARPP32^−^ dataset, and Student *t*-tests were performed to compare the remaining experimental groups.

Additional data for the percentage of c-Fos^+^/GAD67^+^/DARPP32^−^ cell counts were also obtained for the NAc core and shell when the regions were present in the stained tissue (NAc core, Group: wildtype-sham *n* = 5, wildtype-rTMS *n* = 4, ephrin-A2A5^−/−^-sham *n* = 4, and ephrin-A2A5^−/−^-rTMS *n* = 2; NAc shell, Group: wildtype-sham *n* = 6, wildtype-rTMS *n* = 5, ephrin-A2A5^−/−^-sham *n* = 4, and ephrin-A2A5^−/−^-rTMS *n* = 3). This was combined with total c-Fos cells/mm^3^ and percentage c-Fos-DARPP32 cell counts for NAc core and shell from the previous study (Moretti et al., 2021). The percentage of the c-Fos-GAD67 dataset in the NAc core and shell did not meet the assumptions of normality and heterogeneity. Therefore, non-parametric Mann–Whitney *U*-tests were performed to compare the percentage of c-Fos^+^/GAD67^+^/DARPP32^−^ cell counts between sham and treatment for both wildtype and ephrin-A2A5^−/−^ groups.

## 3. Results

### 3.1. Total c-Fos cells/mm^3^

In DLS and DMS, there was no significant strain, treatment, or interaction effects [DLS: F_(1, 16)_ < 1.04, *p* > 0.323; DMS: F_(1, 16)_ < 1.378, *p* > 0.258].

### 3.2. c-Fos colocalization in MSNs

In DLS, there was no overall effect of the strain, treatment, or interaction [F_(1, 16)_ < 0.376, *p* > 0.548] (Figure 2). Similarly, in DMS, there was no significant difference in the percentage of colocalization between strains or treatment [F_(1, 16)_ < 1.26, *p* > 0.279]. However, there was a significant strain^*^treatment interaction [F_(1, 16)_ = 5.1062, *p* = 0.038, η^2^ = 0.228]. To understand the interaction, we followed up with pairwise *post hoc* comparisons using Tukey's test. There was an increase in the percentage of c-Fos-DARPP32 colocalization after treatment in ephrin-A2A5^−/−^ mice compared to the ephrin-sham group (Figure 2); however, the effect did not survive multiple comparison correction [uncorrected *p* = 0.041, t_(16)_ = −2.225, corrected *p* = 0.159] despite the significant omnibus interaction. All other groups were non-significantly different from each other.

### 3.3. Percentage of unspecified c-Fos cells

In DLS and DMS, there was a significant effect of strain, treatment, or interaction [DLS: F_(1, 16)_ < 1.9052, *p* > 0.186; DMS: F_(1, 16)_ < 1.738, *p* > 0.206] (Figure 2).

#### 3.3.1. Confirmatory analysis for unspecified c-Fos-labeled cells

Across all experimental groups, approximately 60 and 40% of c-Fos labeled cells in the ventral and dorsal striatum, respectively, were unidentified (i.e., not MSNs). Therefore, the sections included staining for GABAergic interneurons to identify unspecified c-Fos labeled cells.

For the percentage of c-Fos^+^/GAD67^+^/DARPP32^−^ colocalization, there were no effects of strain, treatment, or interaction for the DLS [F_(1, 14)_ < 0.088, *p* > 0.771], and in the DMS, there was no significant change in c-Fos^+^/GAD67^+^/DARPP32^−^ colocalization between wildtype-sham and wildtype-rTMS mice [t_(9)_ = −0.864, *p* = 0.410], and there was no significant difference between strains, wildtype-sham and ephrin-sham [t_(8)_ = −1.32, *p* = 0.222].

In the NAc core, there was no significant difference in the percentage of c-Fos^+^/GAD67^+^/DARPP32^−^ colocalization following treatment in both wildtype (U = 8.5, *p* = 0.771) and ephrin-A2A5^−/−^ mice (U = 2, *p* = 0.289) and no significant difference between strains, wildtype-sham and ephrin-sham (U = 6, *P* = 0.240). Similarly, in the NAc shell, there was no significant difference in the percentage of c-Fos^+^/GAD67^+^/DARPP32^−^ colocalization following treatment in both wildtype (U = 12, *p* = 0.597) and ephrin-A2A5^−/−^ mice (U = 5, *p* = 0.825). There was also no significant strain difference between wildtype-sham and ephrin-sham (U = 11, *p* = 0.896).

### 3.4. Comparison of c-Fos activity across regions and groups

**Figure 3:**
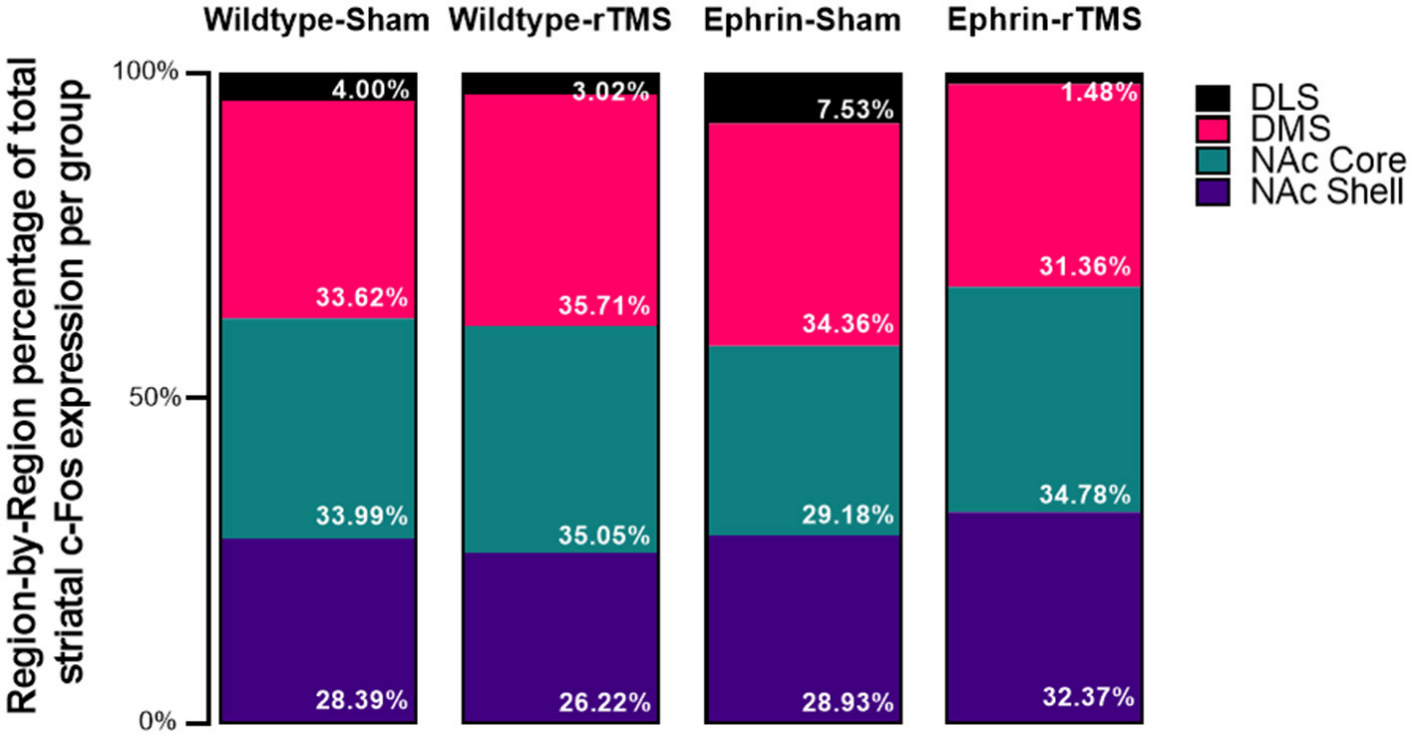
Average region-by-region proportion of the total striatal activity in the experimental groups. An initial 4*4 (group*region) contingency χ^2^ test inclusive of all groups was significant, as were follow-up 2*4 (group*region) contingency χ^2^ tests comparing each experimental group with wildtype-sham. However, Mann–Whitney U comparisons did not identify significant differences at the region level (e.g., wildtype-sham DLS vs. ephrin-sham DLS).

The initial 4^*^4 contingency χ^2^ test was significant [χ^2^(9, N = 666,524) = 8,030, *p* < 0.001, V = 0.0634]. The follow-up 2^*^4 contingency χ^2^ test was performed to understand where the difference was and showed that proportions of the striatal c-Fos activity per region (Figure 3) differed in comparison with wildtype-sham for all groups although the test may be overly sensitive due to the large sample size [wildtype-rTMS: χ^2^(3, N = 436,329) = 642, *p* < 0.001, V = 0.0384; ephrin-sham: χ^2^(3, N = 335,837) = 2156, *p* < 0.001, V = 0.0801; and ephrin-rTMS: χ^2^(3, N = 384,288) = 2446, *p* < 0.001, V = 0.0798]. Although there are significant differences, the effect sizes for each comparison are small.

Finally, a region-by-region comparison of experimental groups against wildtype-sham is presented in Table 1. Despite significant comparisons on the group level, no regions appeared to be statistically different. Therefore, no single regional difference drove the difference between wildtype sham and the other experimental groups. However, numerically, it does appear that the proportion of DLS activation increases in the ephrin-sham group and decreases in the ephrin-rTMS group relative to wildtype mice.

**Table 1:** Region-by-region comparison for the proportion of c-Fos in the striatum.

**Group comparison (wildtype-sham vs. experimental groups)**	**Mann-Whitney U**	***p***	**Effect size (rank biserial correlation)**	**Mean difference**	**95% confidence interval**	
					**Lower**	**Upper**
**Wildtype-rTMS**						
**Region comparison**						
DLS	14	0.926	0.0667	0.271	−2	4
DMS	11	0.537	0.267	−5.5	−22	21
NAc core	14.5	1	0.0333	0.00368	−18	14
NAc shell	12	0.662	0.200	5.5	−11	17
**Ephrin-sham**						
**Region comparison**						
DLS	11.5	0.333	0.361	−3.88	−21	3
DMS	15	0.699	0.167	−5	−24	20
NAc core	11	0.31	0.389	9.5	−9	27
NAc shell	17	0.936	0.056	3	−13	13
**Ephrin-rTMS**						
**Region comparison**						
DLS	4.5	0.276	0.500	2	−2	6
DMS	7	0.714	0.222	4.5	−20	42
NAc core	9	1	0	−0.0727	−23	17
NAc shell	6	0.548	0.333	−4.5	−21	8

Mann–Whitney U comparisons, following significant 2^*^4 contingency χ^2^ tests, assessed region-specific changes in the proportion of striatal c-Fos (wildtype-sham vs. experimental groups).

## 4. Discussion

Prior evidence of a habitual behavioral responding pattern in ephrin-A2A5^−/−^ mice led to the hypothesis that these mice would display greater neuronal activity in striatal regions involved in habit formation (Moretti et al., 2021). The majority of the neuronal activity in the striatum of these mice remained localized in regions responsible for goal-directed behavior. However, the proportion of c-Fos labeling across the striatum was significantly different in all experimental groups compared to control animals although no specific region was identified as driving this difference.

We also hypothesized that the LI-rTMS change in c-Fos densities seen previously in ephrin-A2A5^−/−^ mice NAc would extend to the dorsal striatum. In partial support, we did not observe a change in average c-Fos density after LI-rTMS in ephrin-A2A5^−/−^ mice for the dorsostriatal regions, but in the DMS, there was a trend that suggested increased MSN activation in ephrin-A2A5^−/−^ mice following stimulation.

The proportional activity across the striatum showed significant differences between wildtype-sham and the other experimental groups when considering the distribution of activity across all regions (i.e., wildtype-sham overall proportion distribution vs. wildtype rTMS overall proportion distribution), but when comparing group differences within specific regions (i.e., the difference in NAc proportion between two groups), there were no significant differences. We found absolute differences between the means for the DLS of ephrin-A2A5^−/−^ mice and the wildtype-sham group where proportional DLS activity was greater in ephrin-A2A5^−/−^ sham mice than wildtype mice and lower in ephrin-A2A5^−/−^ mice than LI-rTMS. This is in line with the hypotheses, but the differences did not reach statistical significance. Greater sample size and specific experimental design are required to appropriately understand whether the progression of activity within the striatum is altered with LI-rTMS.

Additionally, we assessed the GABA-ergic interneuron activity throughout the striatum but saw no significant changes in GAD67 activity between groups. Apart from the DMS, which had approximately 5–10% GABA-ergic interneuron activity, low GABA-ergic interneuron activity was observed in the NAc core, shell (< 2%), and DLS (< 4%).

### 4.1. Caveats

The hierarchical recruitment of striatal regions from goal-directed to habitual behavior was partly characterized by the progression of c-Fos expression during training in a fixed-ratio (FR) task, from higher c-Fos activity in the NAc shell on the first day of the task, toward the NAc core, DMS, and finally to DLS as training progresses (Segovia et al., 2012). In our study, a caveat is that, unlike previous studies (Segovia et al., 2012), our c-Fos data indirectly reflect the neuronal activity only at the final timepoint, after the completion of the PR task. In a PR task, unlike in an FR schedule, the response requirement changes over time, so mice cannot generally predict how much work is required to obtain a reward based on previous trials. Although we can assume a similar hierarchical progression of activity within the striatum, the variable nature of the PR task could impact the behavioral and cellular response differently than suggested for the FR framework (Segovia et al., 2012).

## 5. Conclusion

The present study did not fully support the assumptions made by our previous study (Moretti et al., 2021) in which it was hypothesized that the neuronal activity in ephrin-A2A5^−/−^ mice with repetitive and perseverative responding behavior would have shifted to the striatal region involved in habit formation. Rather, in this study, we found that activity remained dominant in regions involved in goal-directed behaviors. However, there was a trend that suggested increased MSN activation in the DMS for ephrin-A2A5^−/−^ following stimulation. The relative proportion of c-Fos activity in the striatum was significantly different in experimental groups compared to the control. However, it is unclear, using the current sample sizes, whether the difference could be statistically attributed to altered dorsal striatum activity.

Although the possibility that rTMS interferes with habit formation is exciting, the current findings remain limited and preliminary. Some of this initial evidence, in addition to recent evidence, for LI-rTMS-induced changes to the striatal activity and network connectivity (Moretti et al., 2022) does provide some direction for potential mechanisms of rTMS involving the modulation of subcortical circuits. This could have implications for the mechanism by which rTMS plays a role in the treatment of conditions such as drug addiction and OCD. Future studies should look at experiments that specifically investigate circuit function and changes over time. Combination with electrophysiological recordings from the striatum could also help map any causal relationship. Overall, LI-rTMS shows some promise for altered hierarchical recruitment of specific striatal regions on a cellular level; however, the current experimental design and sample size limit conclusions. Nonetheless, this evidence supports further circuit-based investigations in larger studies, potentially with more clinically relevant animal models to probe rTMS mechanisms.

## Data availability statement

The raw data supporting the conclusions of this article will be made available by the authors, without undue reservation.

## Ethics statement

The animal study was reviewed and approved by the University of Western Australia Animal Ethics Committee.

## Author contributions

MT: methodology, writing—original draft, writing—review and editing, formal analysis, investigation, and visualization. JR: writing—review and editing and supervision. JM: conceptualization, writing—review and editing, methodology, formal analysis, visualization, and supervision. All authors contributed to the article and approved the submitted version.
